# Effect of needle priming on blood collection time in whole blood donation

**DOI:** 10.6026/973206300210253

**Published:** 2025-02-28

**Authors:** Aaditya Shivhare, Arvind Kumar Singh, Yatendra Mohan, Jyoti Kala Bharati, Nouratan Singh

**Affiliations:** 1 Department of Transfusion Medicine, Uttar Pradesh University of Medical Sciences, Saifai, Etawah, Uttar Pradesh, India

**Keywords:** Citrate-phosphate-adenine (CPDA-1), blood donation protocols, needle priming, enhance efficiency

## Abstract

Efficient and safe blood donation procedures are critical for maintaining an adequate and reliable blood supply. Needle priming, a pre-donation procedure aimed at preventing clot formation, is hypothesized to improve blood flow and reduce donation time. A case-control study was conducted with 340 participants to evaluate the impact of needle priming on whole blood donation. The case group underwent needle priming before donation, while the control group followed standard procedures without priming. The study found a statistically significant reduction in blood collection time in the needle priming group compared to the control group (p < 0.05). Needle priming prior to blood donation significantly enhances procedural efficiency, reduces clotting risks and improves donor satisfaction.

## Background:

Whole blood donation is a critical healthcare practice, supporting millions of transfusions annually to save lives in emergencies, surgeries and chronic illnesses. Despite its importance, prolonged collection times and complications such as clotting can impede the process, affecting donor comfort and blood product quality [1, 2- 3]. Needle priming, where anticoagulant is introduced into the tubing before venipuncture, is a widely accepted practice in apheresis. However, its role in whole blood donation remains under explored [4- 5]. Therefore, it is of interest to evaluate the impact of needle priming on whole blood donation to determine its potential in improving procedural efficiency and reducing complications [6].

## Materials and Methods:

## Study design:

This was a prospective case-control study conducted at the Blood Centre, Uttar Pradesh University of Medical Sciences (UPUMS), Saifai, over 12 months.

## Participants:

## Inclusion criteria:

Healthy blood donors aged 18-60 years, meeting national eligibility criteria [7].

## Exclusion criteria:

Donors with medical conditions affecting blood flow or incomplete data.

## Sample size calculation:

Using an effect size of 15%, significance level α=0.05 and power = 80%, the required sample size was 170 participants in each group (total = 340).

## Procedures:

## Participants were divided into two groups:

[1] Control Group (n=170): Standard blood collection without needle priming.

[2] Case Group (n=170): Blood collection after needle priming with CPDA-1 anticoagulant.

The controls were chosen by matching gender, age, size and time of donation with cases.

Primary outcome: Median blood collection time (seconds).

## Secondary outcomes:

[1] Incidence of clot formation.

[2] Quality of blood products (*e.g.*, PT, INR, Factor VIII levels).

[3] Donor satisfaction, measured via a structured questionnaire.

## Data collection and analysis:

[1] Blood collection time was recorded using a digital stopwatch.

[2] Clotting incidents were documented.

[3] Blood products were analyzed for coagulation parameters using standard laboratory techniques [8- 9].

[4] Statistical analysis was performed using SPSS v25. Continuous variables were analyzed using t-tests or Mann-Whitney U tests and categorical variables with chi-square tests.

## Results:

## Baseline characteristics:

The median age of participants was 30 years (IQR: 25-35), with no significant demographic differences between groups (p > 0.05), as shown in Table 1.

## Primary outcome:

The median blood collection time was significantly shorter in the case group as shown in Table 2. Figure 1 (see PDF) shows the comparison of interquartile ranges of blood collection times between the control and case groups. The case group demonstrates a narrower IQR, indicating more consistent times.

[1] Control Group: 226 seconds (IQR: 202-251)

[2] Case Group: 205 seconds (IQR: 182-239) (p < 0.05, Mann-Whitney U test).

## Key observations:

[1] The case group had a significantly shorter median blood collection time (205 seconds) compared to the control group (226 seconds).

[2] The Interquartile range (IQR) was narrower in the case group (182-239 seconds) than in the control group (202-251 seconds), indicating more consistent collection times.

[3] The p-value (<0.05) signifies a statistically significant difference between the two groups.

## Median blood collection times by group (Figure 1 - see PDF):

[1] X-Axis: Group (Control vs. Case).

[2] Y-Axis: Blood collection time (seconds).

## Secondary outcomes:

##  Clot formation:

**Figure 2:**
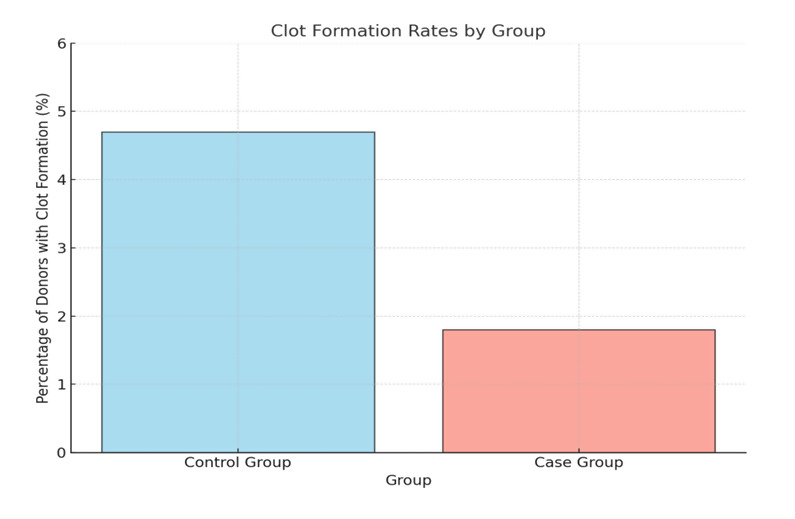
Clot formation rates

Clotting occurred in 4.7% of controls compared to 1.8% of cases (p < 0.05), as shown in Table 3 and Figure 2.

## Observations:

[1] Number of clots: The control group reported 8 clot formation incidents (4.7%), compared to 3 incidents (1.8%) in the case group.

[2] Statistical significance: The p-value (<0.05) indicates that the difference in clot formation incidents between the groups is statistically significant

## Clot formation rates:

[1] X-Axis: Group (Control vs. Case).

[2] Y-Axis: Percentage of donors with clot formation.

## Blood product quality:

Fresh frozen plasma from the primed group exhibited stable coagulation parameters, including PT, INR and Factor VIII levels, as shown in Table 4.

## Observations:

[1] Prothrombin time (PT): Median PT was slightly lower in the case group (13.6 seconds) compared to the control group (13.8 seconds), but the difference was not statistically significant (p=0.34).

[2] International normalized ratio (INR): Both groups had similar INR values (1.02 vs. 1.01), with no significant difference (p=0.29).

[3] Factor VIII (%): Factor VIII levels were marginally higher in the case group (89%) than in the control group (87%), but this difference was not significant (p=0.12).

[4] Fibrinogen: The case group had a slightly higher median fibrinogen level (310 mg/dL) compared to the control group (300 mg/dL), but the difference was not significant (p=0. 18).

[5] Donor satisfaction: 91% of donors in the primed group reported a positive donation experience compared to 78% in the control group (p < 0.01).

## Coagulation parameter stability in FFP:

**Figure 3:**
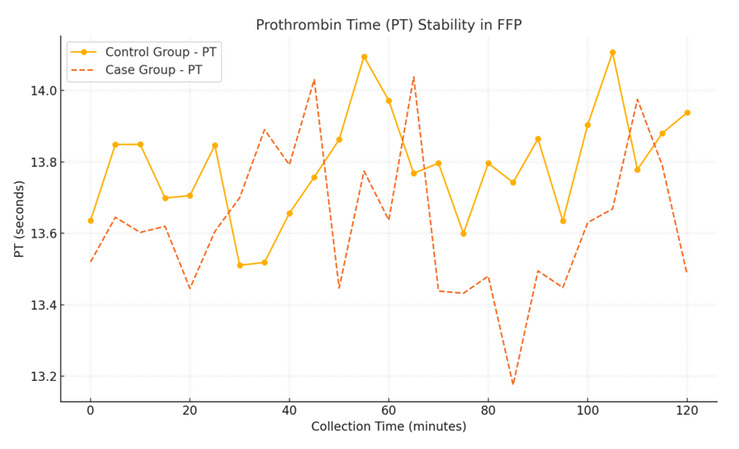
Prothrombin Time (PT) stability in FFP

**Figure 4:**
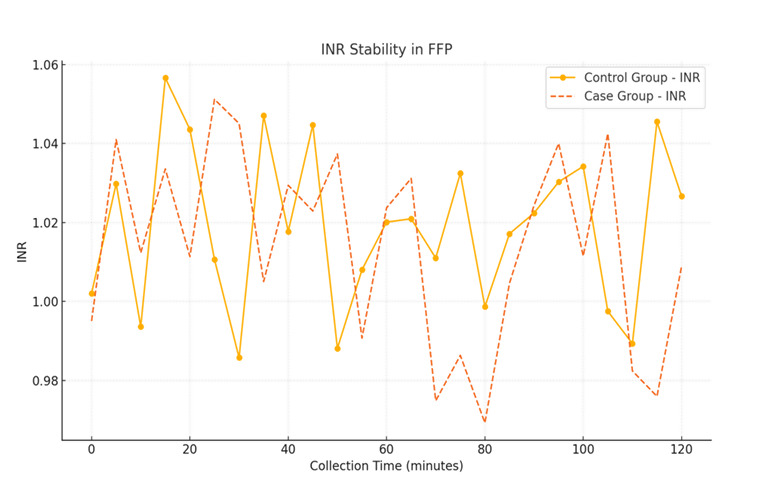
INR stability in FFP

**Figure 5:**
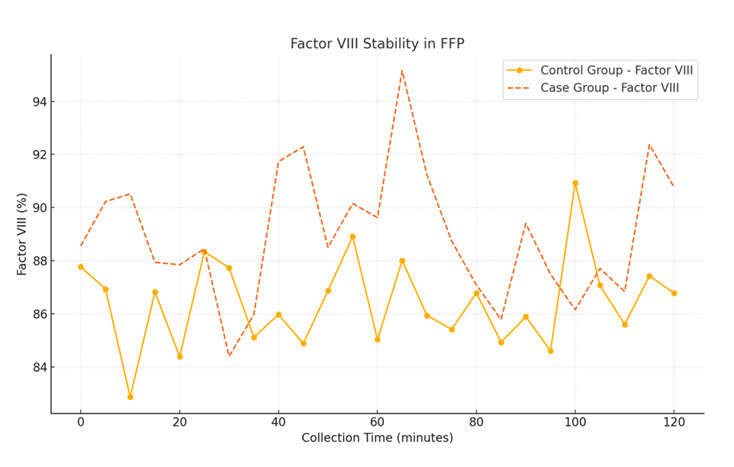
Factor VIII Stability in FFP

A line graph depicting coagulation parameters (PT, INR and Factor VIII) for FFP units collected from both groups. Parameters remain stable across collection times, with no significant deviations in the primed group (Figure 3, Figure 4, Figure 5.

[1] X-Axis: Collection time (minutes).

[2] Y-Axis: Coagulation parameter values.

## Discussion:

This study demonstrates that needle priming significantly reduces blood collection time and clotting incidents, consistent with findings in apheresis procedures [10-11]. By maintaining anticoagulation at the needle tip, priming enhances blood flow, particularly in donors with slower venous return [12]. Improved donor satisfaction highlights the practical benefits of priming, which may encourage repeat donations, crucial for maintaining blood supply [13-14]. Fresh frozen plasma from the primed group maintained consistent coagulation parameters, such as PT, INR and Factor VIII levels, despite extended collection durations. While this study focused on 350 mL collections, future research could explore its applicability to 450 mL donations or specialized populations [15].

## Conclusion:

Anticoagulant priming significantly enhances the efficiency of blood collection processes by reducing collection time and minimizing the risk of clot formation. By preventing initial clotting, which can obstruct blood flow and delay procedures, needle priming optimizes the overall donation experience. The findings underscore the value of integrating priming techniques into routine blood donation protocols to improve procedural efficiency, ensure better sample quality and enhance donor satisfaction. Incorporating these methods can contribute to a more reliable and donor-friendly blood collection system.

## Figures and Tables

**Table 1:** Baseline characteristics of donor

**Characteristic**	**Control group (n=170)**	**Case group (n=170)**	**p-value**
Age (Median, IQR)	30 (25-35)	31 (26-34)	0.55
Gender (Male, %)	98%	97%	0.72
Weight (Kg, Mean ± SD)	69 ±12	70 ±11	0.43
Type of Donor (Replacement, %)	92%	93%	0.65

**Table 2:** Blood collection time comparison

**Group**	**Median time (seconds)**	**IQR**	**p-value**
Control group	226	202-251	
Case group	205	182-239	<0.05

**Table 3:** Clot formation incidents

**Group**	**Clots (n)**	**Percentage (%)**	**p-value**
Control group	8	4.7	<0.05
Case group	3	1.8	

**Table 4:** Quality control of FFP (Median Values)

**Parameter**	**Control Group**	**Case Group**	**p-value**
PT (seconds)	13.8	13.6	0.34
INR	1.02	1.01	0.29
Factor VIII (%)	87	89	0.12
Fibrinogen (mg/dL)	300	310	0.18
